# Clinical epidemiology and associations between HPV infection and vaginal infections in Jinan, China: a cross-sectional analysis

**DOI:** 10.3389/fcimb.2026.1775865

**Published:** 2026-02-27

**Authors:** Xiaodi Chen, Haiyan Zhou, Ruotong Li, Rongguo Li, Peng Liu

**Affiliations:** 1 Department of Clinical Laboratory, Jinan Maternity and Child Care Hospital Affiliated to Shandong First Medical University, Jinan, China; 2 Neonatal Disease Screening Center, Jinan Maternity and Child Care Hospital Affiliated to Shandong First Medical University, Jinan, China

**Keywords:** aerobic vaginitis, bacterial vaginosis, human papillomavirus, *Trichomonas vaginalis*, vaginal infections, vulvovaginal candidiasis

## Abstract

**Background:**

As the principal cause of cervical cancer, human papillomavirus (HPV) infection is linked to vaginal infections such as bacterial vaginosis (BV), vulvovaginal candidiasis (VVC), aerobic vaginitis (AV), and *Trichomonas vaginalis* (TV); however, the exact relationship remains controversial.

**Methods:**

In this study, we performed a cross-sectional analysis of 10,104 women in Jinan, China, to establish a detailed association between HPV and vaginal infections.

**Results:**

Our analysis showed that the HPV infection rate in Jinan was 21.84% of which the high-risk HPV (HR-HPV) infection rate was 84.32%. Although HR-HPV, low-risk HPV, BV, VVC, and TV prevalence rates across seasons were not statistically significant, we discerned significance for AV. In addition, while there was no difference between the prevalence of HPV and VVC, women with BV, AV, TV, or any vaginal infection manifested a higher risk of HPV infection. As for HR-HPV, our results showed statistically significant differences in HR-HPV infection rates between patients with BV, AV, TV, or any type of vaginal infection and the control group; however, VVC cases and cases without VVC did not differ. Furthermore, our correlation analysis among different HR-HPV genotypes and vaginal infections revealed an elevated incidence of BV in individuals with HPV45, HPV51, HPV52, HPV58, HPV66, and HPV68. AV exhibited an elevated infection rate in women with HPV16, HPV33, and HPV68; while TV demonstrated an increased infection risk in women with HPV52.

**Conclusion:**

We hereby explored the complex relationship between HPV infection and vaginal infections and provided information on early-detection, preventive, and therapeutic strategies.

## Introduction

Cervical cancer currently ranks fourth in incidence and mortality rates globally among cancers in women, with 661,021 new cases and 348,189 deaths reported worldwide in 2022 (Bray et al., 2024). Furthermore, there were 150,659 newly diagnosed cases of cervical cancer and 55,694 deaths attributed to the disease in China in 2022 (Bray et al., 2024; Ma et al., 2025). Cervical cancer is consequently a major global health concern for Chinese women (Xu et al., 2024). As a circular, non-enveloped deoxyribonucleic acid virus, human papillomavirus (HPV) is a major cause of cervical cancer (Evans et al., 2022; Wei et al., 2024; Zhang Z. et al., 2024), with more than 400 HPV genotypes having been identified and with roughly 40 of these infecting the vaginal tract (Van Doorslaer et al., 2017). The HPV genotypes related to reproductive tract infections are divided into two groups: high-risk HPV (HR-HPV) and low-risk HPV (LR-HPV) (Zou et al., 2022). Cervical cancer is internationally acknowledged to be caused by HR-HPV infection, and mild squamous epithelial lesions and genitourinary warts are associated with LR-HPV (Hebner and Laimins, 2006; McBride, 2022).

The vaginal microbiota is a unique element of the human microbiome and comprises a diverse community of microorganisms (Lebeer et al., 2023; Liu P. et al., 2023). This vaginal microenvironment is typically dominated by *Lactobacillus* spp. that can acidify the environment, reduce the proliferation of other pathogenic microorganisms, and facilitate the preservation of a eubiotic vaginal microbiota (Norenhag et al., 2020; Lee et al., 2023). However, vaginal flora with a reduced proportion or abundance of lactobacilli can lead to vaginal dysbiosis, which, in turn, is associated with vaginal infections such as bacterial vaginosis (BV), vulvovaginal candidiasis (VVC), aerobic vaginitis (AV), and *Trichomonas vaginalis* (TV) (Liu P. et al., 2023; Muzny et al., 2023). These vaginal infections can result in serious health consequences that include infertility, preterm birth, pelvic inflammatory disease, premature rupture of membranes, and miscarriage (Plisko et al., 2021; Harfouche et al., 2024; Bradshaw et al., 2025; Valentine et al., 2025).

Prior research has suggested that the relationship between abnormal vaginal flora and HPV infection in women remains controversial. Some researchers found that the variety of microbiota was much higher in women who had HPV than in women who did not have HPV, and they also ascertained that having HPV was closely linked to greater abundances of *Gardnerella* and *Prevotella* and less of *Lactobacillus* species (Romero-Morelos et al., 2019; Cheng et al., 2020; Lin et al., 2022). However, some authors have drawn differing conclusions (Rahkola et al., 2009; Hu et al., 2021). Rahkola et al. observed that the infection rate with BV exhibited no significant difference between women who were HR-HPV positive and those who were HR-HPV negative (Rahkola et al., 2009). Similarly, Hu et al. reported that the persistence of HPV infection (excluding HPV16 and HPV18 genotypes) among women was not associated with common vaginal infections (Hu et al., 2021), regardless of whether they had received the HPV16/18 vaccine. Importantly, the investigators who led the studies noted above solely examined the differences in vaginal infections between HPV-positive women and HPV-negative women; thus, the association between vaginal infections and detailed HPV genotypes necessitates further study.

To address the aforementioned issues, we performed a cross-sectional analysis to assess the association between HPV infection and vaginal infections that included BV, AV, VVC, and TV. We thereby retrospectively examined the prevalence and genotypic distribution of HPV infection among women in Jinan, Shandong Province, China; and we analyzed the relationship between HPV infection-particularly various HPV genotypes-and vaginal infections. We posit that our research findings will provide a more rigorous theoretical foundation for the prevention, evaluation, and follow-up of HPV-positive patients who also possess related infectious diseases.

## Materials and methods

### Data sources

We conducted a cross-sectional study on women who attended the Jinan Maternity and Child Care Hospital from June 1, 2019, to May 31, 2022. Within this timeframe, 187,027 women underwent microecologic analyses of their reproductive tracts, and 37,494 women underwent HPV genotyping of their cervical secretions. A total of 10,104 patients who underwent both reproductive tract microecology testing and cervical secretion HPV genotyping were screened out and enrolled for subsequent analysis. All of our selected patients met the following criteria: (1) they had not undergone vaginal douching or receive intravaginal medication within 3 days prior to sample collection; (2) they did not have sexual intercourse within 24 hours before the test; (3) their samples were collected outside of their menstrual period; and (4) they had not applied acetic acid or iodine solution during the HPV testing process. This study was approved by the Medical Ethics Committee of the Jinan Maternity and Child Care Hospital Affiliated with Shandong First Medical University (IRB KY-23-55).

### Methods

#### Sample collection

(1) For the collection of vaginal microecology samples, we used a disposable, clean, and dry cotton swab or suction device to collect a sufficient amount of secretion from the posterior fornix of the vagina. The sample was then placed in a disposable plastic vial. (2) For the collection of HPV test samples, we employed a cervical exfoliated cell collector. Healthcare professionals applied a speculum or vaginal dilator to expose the cervix and wiped off excess cervical mucus using a cotton swab. The cervical brush was then inserted into the cervical os and rotated four to five times in one direction to collect an adequate epithelial cell sample. The brush head was then placed into the elution tube, and the brush handle was broken off at the marked point. The elution tube was sealed tightly, labeled, and stored upright.

### Vaginal microecology testing

(1) A suspension of the sample was prepared by adding six to eight drops of diluent to a glass slide. A wet mount was then prepared and observed under a low-power microscope for the presence of fungal hyphae and *Trichomonas vaginalis*. (2) A drop of the sample suspension was placed at the center of the slide, dried, fixed, and Gram-stained. We observed the presence of fungal hyphae under high magnification, whereas bacterial flora was assessed under oil immersion. The results were interpreted using the Nugent (Nugent et al., 1991) and AV scoring systems (Donders et al., 2002).

### HPV genotyping testing

We conducted HPV genotyping using the HPV Genotyping Kit (24 types) (Beijing Bohui Innovation Biotechnology Co., Ltd., Beijing, China; Catalog No. BH-HPV24-96) according to the manufacturer’s instructions. This kit can identify 24 HPV genotypes composed of 18 HR-HPV types (HPV16, HPV18, HPV31, HPV33, HPV35, HPV39, HPV45, HPV51, HPV52, HPV53, HPV56, HPV58, HPV59, HPV66, HPV68, HPV73, HPV82, and HPV83) and six LR-HPV types (HPV6, HPV11, HPV42, HPV43, HPV44, and HPV81). Our testing method combined PCR amplification and DNA reverse blot hybridization using DNA chip technology. The procedure included several crucial steps: initially, HPV DNA was isolated from the clinical specimen, and the collected DNA was amplified utilizing the appropriate primers. These primers were intended to target a conserved region of the L1 gene across a wide array of HPV genotypes. The PCR products were tagged with biotin during amplification. Subsequently, the labelled PCR products were denatured and hybridized to a DNA microarray chip. This chip featured numerous tiny regions, each housing a distinct immobilized DNA probe tailored to a specific HPV genotype. Third, the chip was treated with a streptavidin-horseradish peroxidase combination. Streptavidin exhibited a significant affinity for biotin. A subsequent incubation with a Tetramethylbenzidine substrate solution resulted in the formation of a blue precipitate only at the sites of hybridization.

### Statistical analysis

We adopted SPSS statistical software 23.0 (IBM, Chicago, USA) to analyze our study data. Categorical data are expressed as frequencies (*n*) and percentages (%), with non-ranked categorical data analyzed with the *χ*^2^ test or Fisher’s exact test. Odds ratios (ORs) and their 95% confidence intervals (CIs) were calculated to describe the risk factors associated with HPV infection. A *p* value of <0.05 was considered to be statistically significant.

## Results

### HPV infection overview

#### HPV infection rate

**Figure 1:**
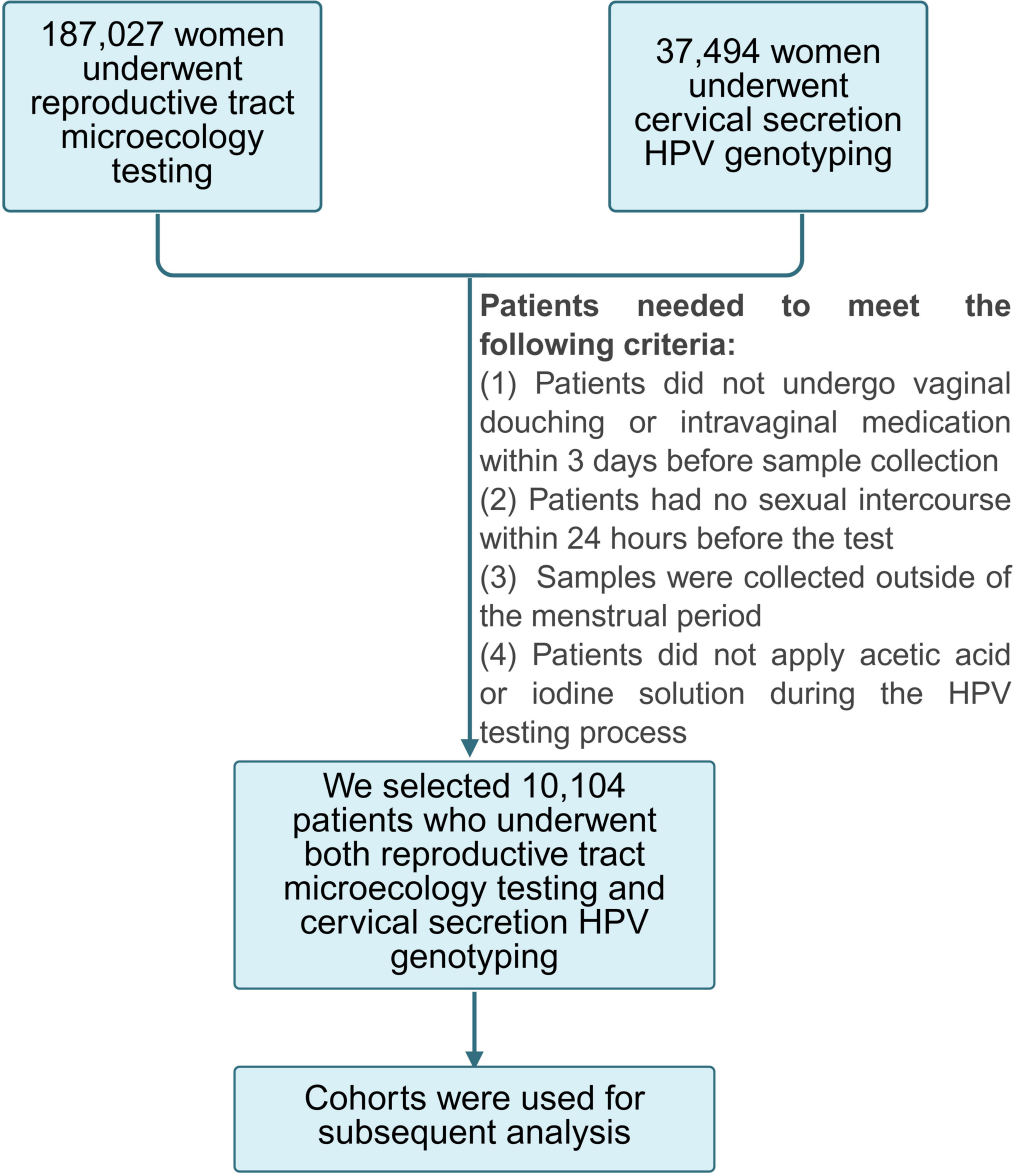
Study flowchart. A total of 10,104 patients who underwent both microecology testing of their reproductive tracts and HPV genotyping of their cervical secretions were screened out and enrolled for subsequent analysis.

This study cohort consisted of 10,104 women who underwent both reproductive tract microecology testing and cervical secretion HPV genotyping (**Figure 1**), with a mean age of the participants at 36.44 ± 9.92 years. Of the 10,104 women included in this study, 2,207 tested positive for HPV, resulting in an infection rate of 21.84% (2,207/10,104). Among the HPV-positive patients, 1,488 cases (67.42%, 1,488/2,207) had a single genotype infection, whereas 719 cases (32.58%, 719/2,207) manifested multiple genotype infections, including 488 cases (22.11%, 488/2,207) with dual genotype infections, 146 cases (6.62%, 146/2,207) with triple genotype infections, and 85 cases (3.85%, 85/2,207) with over three genotype infections. Four cases exhibited infection with up to nine genotypes.

#### Genotypes of HPV infection in 2,207 HPV-positive women

We uncovered 24 different HPV genotypes in the 2,207 HPV-positive women, including 18 HR-HPV types (HPV16, HPV18, HPV31, HPV33, HPV35, HPV39, HPV45, HPV51, HPV52, HPV53, HPV56, HPV58, HPV59, HPV66, HPV68, HPV73, HPV82, and HPV83) and six LR-HPV types (HPV6, HPV11, HPV42, HPV43, HPV44, and HPV81). An infection was considered to be HR-HPV if at least one HR-HPV genotype was present. HR-HPV infections accounted for 84.32% (1,861/2,207) of all HPV infections, whereas LR-HPV infections accounted for 15.68% (346/2,207). We noted single HR-HPV infections in 52.88% (1,167/2,207) of cases; as the two most concerning HR-HPV genotypes, single HPV16 infection was detected in 12.43% (145/1,167) of single HR-HPV infections, while single HPV18 infection accounted for 2.40% (28/1,167) of these cases. The six most frequently detected single HR-HPV subtypes were HPV 52 (19.97%, 233/1167), HPV 16 (12.43%, 145/1167), HPV 58 (11.05%, 129/1167), HPV 51 (7.20%, 84/1167), HPV 53 (7.03%, 82/1167), and HPV 66 (6.26%, 73/1167) (the detection rates of each HPV subtype are listed in **Table 1**).

**Table 1:** Genotype distribution of 1,167 single HR-HPV infections among 1,861 HR-HPV–infected women.

HR-HPV genotype	Frequency (*n*)	Ratio (%)
HPV 52	233	19.97 (233/1,167)
HPV 16	145	12.43 (145/1,167)
HPV 58	129	11.05 (129/1,167)
HPV 51	84	7.20 (84/1,167)
HPV 53	82	7.03 (82/1,167)
HPV 66	73	6.26 (73/1,167)
HPV 56	70	6.00 (70/1,167)
HPV 39	65	5.57 (65/1,167)
HPV 68	65	5.57 (65/1,167)
HPV 59	50	4.28 (50/1,167)
HPV 31	36	3.08 (36/1,167)
HPV 33	33	2.83 (33/1,167)
HPV 35	33	2.83 (33/1,167)
HPV 18	28	2.40 (28/1,167)
HPV 82	22	1.89 (22/1,167)
HPV 45	15	1.29 (15/1,167)
HPV 73	4	0.34 (4/1,167)

HR-HPV, high-risk human papillomavirus.

#### Age distribution of HPV infections

**Figure 2:**
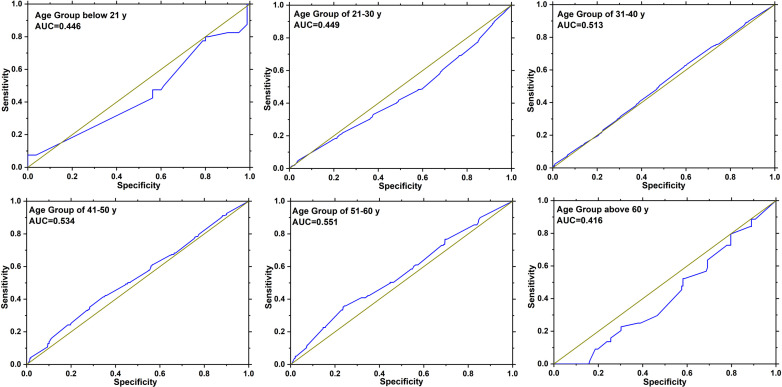
ROC curves and AUC values for HR-HPV infection in age-specific groups. The deep yellow curve represents random chance, and the blue curve represents the sensitivity curve; the closer the curve was to the upper left, the more accurate the analysis was. This figure was generated by using SPSS software 23.0.

As presented in **Table 2**, a majority of the 10,104 patients were between the ages of 21 and 50, comprising 88.14% (8,906/10,104) of the total cohort and contributing to 87.54% (1,932/2,207) of all HPV infections. There was a statistically significant difference in HPV infection among the different age groups (*p* < 0.01): HPV infection rates were the highest among patients younger than 21 years (44.17%, 53/120) and the lowest among those patients aged 21–30 years (26.21%, 742/2,831). Furthermore, in this study, the highest HR-HPV infection rate was observed in the <21 age group (33.33%, 40/120), and the lowest HR-HPV infection rate was found in the 41–50 age group (13.64%, 227/1,664). As depicted in **Figure 2**, we determined the receiver operating characteristic (ROC) curves and the areas under the ROC curve (AUCs) for HR-HPV infection together with the age-specific groups and demonstrated that the AUC values for each age-specific group were 0.446, 0.449, 0.513, 0.534, 0.551, and 0.416, respectively.

**Table 2:** HPV prevalence in various age groups among 10,104 women.

Age groups (y)	HPV-positive			HPV-negative (*%*)	Total (*n*)	*χ* ^2^	*p*
	HR-HPV (*%*)	LR-HPV(*%*)	Total (%)				
Below 21	33.33 (40/120)	10.83 (13/120)	44.16 (53/120)	55.83 (67/120)	120	90.75	0.000
21–30	22.22 (629/2,831)	3.99 (113/2,831)	26.21 (742/2,831)	73.79 (2,089/2,831)	2,831		
31–40	17.27 (762/4,411)	3.04 (134/4,411)	20.31 (896/4,411)	79.69 (3,515/4,411)	4,411		
41–50	13.64 (227/1,664)	4.03 (67/1,664)	17.67 (294/1,664)	82.33 (1,370/1,664)	1,664		
51–60	18.45 (159/862)	1.97 (17/862)	20.42 (176/862)	79.58 (686/862)	862		
Above 60	20.37 (44/216)	0.93 (2/216)	21.30 (46/216)	78.70 (170/216)	216		

HR-HPV, high-risk human papillomavirus; LR-HPV, low-risk human papillomavirus.

### Frequency and prevalence of HPV infection and vaginal infections in different seasons

Based on the China Meteorological Information Center’s standard QX/T152-2012 (data.cma.cn), we subdivided the months into four groups: Spring (March-May), Summer (June-August), Autumn (September-November), and Winter (December-February of the following year). As shown in **Table 3**, the seasonal distributions of HR-HPV, LR-HPV, BV, AV, VVC, and TV were analyzed in the 10,104 women, and we detected variation in the rates of dysbiosis across different seasons. The prevalences of AV in spring, summer, autumn, and winter were significantly different at 7.09% (198/2,791), 4.48% (123/2,573), 5.19% (144/2,775), and 6.21(122/1,965), respectively (*p* < 0.05). The prevalences of HR-HPV, LR-HPV, BV, VVC, and TV were highest in winter (19.95%, 392/1,965), spring (3.80%, 106/2,791), autumn (14.38%, 399/2,775), winter (14.45%, 284/1,965), and summer (0.89%, 23/2,573), respectively; and lowest in spring (17.81%, 497/2,791), autumn (3.06%, 85/2,775), spring (12.90%, 360/2,791), summer (12.75%, 328/2,573), and winter (0.76%, 15/1,965), respectively. However, the prevalences of HR-HPV, LR-HPV, BV, VVC, and TV did not differ across seasons (*p* > 0.05).

**Table 3:** Frequency and prevalence of HPV infection and vaginal infections during the various seasons of the year.

Season/months	Sample size (*n*)	HPV-positive		BV (%)	AV (%)	VVC (%)	TV (%)
		HR-HPV (%)	LR-HPV (%)				
Spring (March-May)	2,791	17.81 (497/2,791)	3.80 (106/2,791)	12.90 (360/2,791)	7.09 (198/2,791)	13.87 (387/2,791)	0.79 (22/2,791)
Summer (June-August)	2,573	18.31 (471/2,573)	3.23 (83/2,573)	14.11 (363/2,573)	4.48 (123/2,573)	12.75 (328/2,573)	0.89 (23/2,573)
Autumn (September-November)	2,775	18.05 (501/2,775)	3.06 (85/2,775)	14.38 (399/2,775)	5.19 (144/2,775)	13.95 (387/2,775)	0.79 (22/2,775)
Winter (December-February)	1,965	19.95 (392/1,965)	3.66 (72/1,965)	13.89 (273/1,965)	6.21 (122/1,965)	14.45 (284/1,965)	0.76 (15/1,965)
*χ* ^2^		4.03	2.92	2.91	15.92	3.12	0.30
*p*		0.259	0.404	0.407	0.001	0.374	0.959

HR-HPV, high-risk human papillomavirus; LR-HPV, low-risk human papillomavirus; BV, bacterial vaginosis; AV, aerobic vaginitis; VVC, vulvovaginal candidiasis; and TV, *Trichomonas vaginalis*.

### Evaluation of the correlations of vaginal infections with HPV infection

#### Analysis of the correlations between HPV infection and BV, AV, VVC, and TV

BV was detected in 1,395 of the 10,104 cases, comprising 13.81% (1,395/10,104) of the cohort. In addition, AV was found in 5.81% (587/10,104) of cases, VVC in 13.72% (1,386/10,104), and TV in 0.81% (82/10,104). Patients with any type of BV, AV, VVC, or TV accounted for 29.49% (2,980/10,104). Patients who had not been diagnosed with BV, AV, VVC, TV, or any form of vaginal infection were adopted as the control group, and the association between HPV infection rate and BV, AV, VVC, TV, or any type of vaginal infection rates was analyzed. As presented in **Table 4**, our results revealed statistically significant differences in HPV infection rates between patients with BV, intermediate BV, AV, TV, or any type of vaginal infection and the control group (*p* < 0.05). However, VVC cases and cases without VVC were not statistically different (*p* > 0.05). Furthermore, the risk of HPV infection was higher in patients with BV (OR, 1.95; 95% CI, 1.72–2.21), AV (OR, 1.33; 95% CI, 1.10–1.61), TV (OR, 1.97; 95% CI, 1.25–3.11), or with any form of vaginal infection (OR, 1.45; 95% CI, 1.32–1.61); whereas women with HPV infection had a lower risk of intermediate BV (OR, 0.86; 95% CI, 0.76–0.97) than women without BV infection.

**Table 4:** Association between vaginal infections and HPV infection.

Vaginal infection	No. of visits (*n*)	Ratio of visits with HPV infection (%)	Ratio of visits without HPV infection (%)	OR	95% CI	*χ* ^2^	*p*
BV							
No (Nugent score 0–3)	6,483	20.58 (1,334/6,483)	79.42 (5,149/6,483)	Ref	Ref	Ref	Ref
Intermediate BV (Nugent score 4–6)	2,226	18.19 (405/2,226)	81.81 (1,821/2,226)	0.86	0.76–0.97	5.89	0.015
BV (Nugent score 7–10)	1,395	33.55 (468/1,395)	66.45 (927/1,395)	1.95	1.72–2.21	109.49	0.000
AV							
No (AV score <3)	9,517	21.54 (2,050/9,517)	78.46 (7,467/9,517)	Ref	Ref	Ref	Ref
Yes (AV score ≥3)	587	26.75 (157/587)	73.25 (430/587)	1.33	1.10–1.61	8.78	0.003
VVC							
No	8,718	21.83 (1,903/8,718)	78.17 (6,815/8,718)				
Yes	1,386	21.93 (304/1,386)	78.07 (1,082/1,386)	1.01	0.88–1.15	0.01	0.931
TV							
No	10,022	21.73 (2,178/10,022)	78.27 (7,844/10,022)	Ref	Ref	Ref	Ref
Yes	82	35.37 (29/82)	64.63 (53/82)	1.97	1.25–3.11	8.86	0.003
Any type of vaginal infection with BV, AV, VVC, and/or TV							
No	7,124	19.89 (1,417/7,124)	80.11 (5,707/7,124)	Ref	Ref	Ref	Ref
Yes	2,980	26.51 (790/2,980)	73.49 (2,190/2,980)	1.45	1.32–1.61	53.93	0.000

No., number; HPV, human papillomavirus; OR, odds ratio; 95% CI, 95% confidence interval; Ref, reference group; BV, bacterial vaginosis; AV, aerobic vaginitis; VVC, vulvovaginal candidiasis; and TV, *Trichomonas vaginalis*.

#### Analysis of the correlation between HR-HPV infection and BV, AV, VVC, and TV

We next investigated the correlation between HR-HPV infection and BV, AV, TV, and VVC. Patients who had not been diagnosed with BV, AV, VVC, TV, or any form of vaginal infection were used as the control group. We analyzed the association between HR-HPV infection and BV, AV, VVC, TV, or any type of vaginal infection. **Table 5** results show statistically significant differences in HR-HPV infection rates between patients with BV, AV, TV, or any type of vaginal infection and the control group (*p* < 0.05); however, VVC cases and cases without VVC did not differ (*p* > 0.05). These findings were consistent with the association between HPV infection and TV, BV, AV, and VVC. In contradistinction, the HR-HPV infection rates between cases with intermediate BV and cases without BV were not statistically different *(p* > 0.05), which was not consistent with a potential association between HPV infection and intermediate BV. Furthermore, the risk of HR-HPV infection was higher in patients with BV (OR, 2.04; 95% CI, 1.79–2.33), AV (OR, 1.39; 95% CI, 1.14–1.69), TV (OR, 1.96; 95% CI, 1.22–3.14), or any type of vaginal infection (OR, 1.48; 95% CI, 1.33–1.64); whereas the women with HR-HPV infection manifested a lower risk of intermediate BV (OR, 0.89; 95% CI, 0.78–1.02) relative to women without BV infection.

**Table 5:** Association between vaginal infections and HR-HPV infection.

Vaginal infection	No. of visits (*n*)	Ratio of visits with HR-HPV infection (%)	Ratio of visits without HR-HPV infection (%)	OR	95% CI	*χ* ^2^	*p*
BV							
No (Nugent score 0–3)	6,483	17.04 (1,105/6,483)	82.96 (5,378/6,483)	Ref	Ref	Ref	Ref
Intermediate BV (Nugent score 4–6)	2,226	15.45 (344/2,226)	84.55 (1,882/2,226)	0.89	0.78–1.02	3.02	0.082
BV (Nugent score 7–10)	1,395	29.53 (412/1,395)	70.47 (983/1,395)	2.04	1.79–2.33	115.17	0.000
AV							
No (AV score <3)	951	18.10 (1,723/9,517)	81.90 (7,794/9,517)	Ref	Ref	Ref	Ref
Yes (AV score ≥3)	587	23.51 (138/587)	76.49 (449/587)	1.39	1.14–1.69	10.75	0.001
VVC							
No	8,718	18.49 (1,612/8,718)	81.51 (7,106/8,718)	Ref	Ref	Ref	Ref
Yes	1,386	17.97 (249/1,386)	82.03 (1,137/1,386)	0.97	0.83–1.12	0.22	0.639
TV							
No	10,022	18.32 (1,836/10,022)	81.68 (8,186/10,022)	Ref	Ref	Ref	Ref
Yes	82	30.49 (25/82)	69.51 (57/82)	1.96	1.22–3.14	8.01	0.005
Any type of vaginal infection with BV, AV, VVC, and/or TV							
No	7,124	16.61 (1,183/7,124)	83.39 (5,941/7,124)	Ref	Ref	Ref	Ref
Yes	2,980	22.75 (6,78/2,980)	77.25 (2,302/2,980)	1.48	1.33–1.64	52.82	0.000

No., number; HR-HPV, high-risk human papillomavirus; OR, odds ratio; 95% CI, 95% confidence interval; Ref, reference group; BV, bacterial vaginosis; AV, aerobic vaginitis; VVC, vulvovaginal candidiasis; and TV, *Trichomonas vaginalis*.

#### Analysis of the correlation between each HR-HPV genotype and BV, AV, VVC, and TV

The prevalences of BV, TV, VVC, and AV among different HR-HPV subtypes are depicted in **Table 6**. Correlation analysis of various HR-HPV genotypes with BV revealed that the risk of BV infection was higher in patients with HPV45 (OR, 3.33; 95% CI, 1.14–9.75), HPV51 (OR, 1.96; 95% CI, 1.17–3.27), HPV52 (OR, 1.73; 95% CI, 1.25–2.39), HPV58 (OR, 2.14; 95% CI, 1.42–3.21), HPV66 (OR, 2.03; 95% CI, 1.17–3.50), and HPV68 (OR, 2.00; 95% CI, 1.12–3.57). AV also exhibited a higher infection rate in women with HPV16 (OR, 1.97; 95% CI, 1.15–3.39), HPV33 (OR, 3.02; 95% CI, 1.16–7.84), or HPV68 (OR, 2.37; 95% CI, 1.13–5.00); while TV demonstrated a higher infection rate in women with HPV 52 (OR, 3.78; 95% CI, 1.62–8.81). However, VVC exhibited no significant correlation with any of the HR-HPV genotypes that we analyzed.

**Table 6:** Association between vaginal infections and specific HR-HPV genotype.

HR-HPV genotype	BV				AV			
	Ratio (%)	OR	95% CI	*p*	Ratio (%)	OR	95% CI	*p*
HPV 16	17.2 (25/145)	1.40	0.90–2.16	0.131	10.3 (15/145)	1.97	1.15–3.39	0.012
HPV 18	17.9 (5/28)	1.44	0.55–3.80	0.404	3.6 (1/28)	0.62	0.09–4.60	1.000
HPV 31	22.2 (8/36)	1.91	0.87–4.19	0.130	8.3 (3/36)	1.54	0.47–5.02	0.453
HPV 33	21.2 (7/33)	1.19	0.78–4.13	0.190	15.2 (5/33)	3.02	1.16–7.84	0.036
HPV 35	18.2 (6/33)	1.48	0.61–3.59	0.432	3 (1/33)	0.53	0.07–3.86	1.000
HPV 39	10.8 (7/65)	0.80	0.36–1.75	0.575	9.2 (6/65)	1.72	0.74–3.99	0.180
HPV 45	33.3 (5/15)	3.33	1.14–9.75	0.037	6.7 (1/15)	1.20	0.16–9.17	0.579
HPV 51	22.6 (19/84)	1.96	1.17–3.27	0.009	4.8 (4/84)	0.85	0.31–2.32	1.000
HPV52	20.2 (47/233)	1.73	1.25–2.39	0.001	7.3 (17/233)	1.34	0.81–2.22	0.248
HPV 53	19.5 (16/82)	1.62	0.94–2.81	0.082	6.1 (5/82)	1.10	0.44–2.72	0.807
HPV 56	15.7 (11/70)	1.23	0.65–2.36	0.523	0	–	–	1.000
HPV 58	24.0 (31/129)	2.14	1.42–3.21	0.000	7.0 (9/129)	1.26	0.64–2.50	0.503
HPV 59	22.0 (11/50)	1.88	0.96–3.69	0.061	8.0 (4/50)	1.48	0.53–4.12	0.360
HPV 66	23.3 (17/73)	2.03	1.17–3.50	0.010	5.5 (4/73)	0.98	0.35–2.68	1.000
HPV 68	23.1 (15/65)	2.00	1.12–3.57	0.017	12.3 (8/65)	2.37	1.13–5.00	0.019
HPV 73	0	–	–	1.000	25.0 (1/4)	5.63	0.58–54.16	0.206
HPV 82	13.6 (3/22)	1.04	0.31–3.51	1.000	0	–	–	1.000
HR-HPV genotype	VVC				TV			
	Ratio (%)	OR	95% CI	*p*	Ratio (%)	OR	95% CI	*p*
HPV 16	17.9 (26/145)	1.42	0.93–2.18	0.106	1.4 (2/145)	1.88	0.46–7.75	0.295
HPV 18	17.9 (5/28)	1.41	0.53–3.70	0.414	0	–	–	1.000
HPV 31	8.3 (3/36)	0.59	0.18–1.91	0.470	0	–	–	1.000
HPV 33	15.2 (5/33)	1.16	0.45–3.00	0.796	0	–	–	1.000
HPV 35	6.1 (2/33)	0.42	0.10–1.74	0.306	0	–	–	1.000
HPV 39	13.8 (9/65)	1.03	0.51–2.09	0.929	1.5 (1/65)	1.68	0.23–12.22	0.456
HPV 45	6.7 (1/15)	0.46	0.06–3.52	0.709	0	–	–	1.000
HPV 51	11.9 (10/84)	0.87	0.45–1.69	0.680	1.2 (1/84)	1.60	0.22–11.65	0.471
HPV 52	9.9 (23/233)	0.70	0.45–1.08	0.105	2.6 (6/233)	3.78	1.62–8.81	0.007
HPV 53	13.4 (11/82)	1.00	0.53–1.89	0.999	0	–	–	1.000
HPV 56	15.7 (11/70)	1.20	0.63–2.29	0.577	0	–	–	1.000
HPV 58	12.4 (16/129)	0.91	0.54–1.54	0.719	1.6 (2/129)	2.14	0.52–8.80	0.249
HPV 59	8.0 (4/50)	0.56	0.20–1.56	0.402	0	–	–	1.000
HPV 66	16.4 (12/73)	1.28	0.69–2.38	0.440	0	–	–	1.000
HPV 68	16.9 (11/65)	1.32	0.69–2.52	0.408	1.5 (1/65)	2.09	0.29–15.23	0.388
HPV 73	50.0 (2/4)	6.48	0.91–46.05	0.089	0	–	–	1.000
HPV 82	13.6 (3/22)	1.02	0.30–3.45	1.000	0	–	–	1.000

HR-HPV, high-risk human papillomavirus; OR, odds ratio; 95% CI, 95% confidence interval; BV, bacterial vaginosis; AV, aerobic vaginitis; VVC, vulvovaginal candidiasis; TV, *Trichomonas vaginalis*; and -, no results.

## Discussion

In November of 2020, the World Health Organization (WHO) launched a strategy to reduce HPV incidence worldwide from 13.3 per 100,000 women to 4 per 100,000 by 2030, with the aim of completely eliminating cervical cancer (Shen et al., 2023). In response to this appeal by the WHO, the National Health Commission of China has been implementing a program to screen Chinese women for cervical cancer, setting a target of achieving over 50% screening coverage among women aged 35–64 by the end of 2025 (Zhang et al., 2021). Because persistent infection with HR-HPV constitutes a significant factor in the etiology of cervical cancer, an assessment of the epidemiology of HPV infection is urgently needed. The HPV infection rate was 21.84% in Jinan in our analysis, which was higher than that reported in previous studies for other regions of China, including Hengyang (10.16%) (Tang et al., 2021), Tianjin (14.71%) (Chen X. et al., 2015), Xiamen (15.13%) (Shen et al., 2023), Shanghai (17.92%) (Li et al., 2020), Yueyang (20.47%) (Hou et al., 2023), and Guangzhou (21.66%) (Yang et al., 2023). Nevertheless, Jinan exhibited a lower HPV prevalence than Hangzhou (22.41%) (Wang et al., 2021), Luoyang (22.81%) (Wang et al., 2022), Chengdu (23.38%) (Zhang J. et al., 2024), Weifang (23.64%) (Liu M. et al., 2023), Chongqing (26.2%) (Tang et al., 2017), Changsha (26.54%) (Xiao et al., 2016), and Xi’an (30.21%) (Cao et al., 2017). The distribution patterns of HPV genotypes were also diverse across different regions in China. In our investigation, HPV52 emerged as the predominant genotype in HPV-positive patients, and the same results were also obtained in Xiamen (Shen et al., 2023), Chengdu (Zhang J. et al., 2024), Guangzhou (Yang et al., 2023), Shanghai (Li et al., 2020), Yueyang (Hou et al., 2023), and Changsha (Xiao et al., 2016). However, in Hangzhou (Wang et al., 2021), Luoyang (Wang et al., 2022), Hengyang (Tang et al., 2021), Tianjin (Chen X. et al., 2015), Xi’an (Cao et al., 2017), and Weifang (Liu M. et al., 2023), HPV16 was the predominant HPV genotype. There were, additionally, variations in the prevalence of HPV infection among age groups. Our findings revealed that the HPV infection rate was highest among patients under 21 years of age, congruent with studies from Xiamen (Shen et al., 2023) and Chengdu (Zhang J. et al., 2024). Conversely, in Changsha (Xiao et al., 2016), Yueyang (Hou et al., 2023), Xi’an (Cao et al., 2017), and Hengyang (Tang et al., 2021), the oldest age group demonstrated the highest HPV rate. Numerous factors such as age, ethnicity, geographical settings, and socioeconomic level may account for the substantial disparities in HPV infection rates across various regions in China (Duan et al., 2020; Han et al., 2025).

Previous studies revealed seasonal variation in the incidence of VVC and HPV infections (Chen et al., 2019; Donders et al., 2022). For example, a study of vaginal cultures conducted over a decade in a non-selected population showed seasonal variations in the frequency of VVC, with augmented rates during summer and diminished rates in winter (Donders et al., 2022). Chen et al. also discovered that the positive rate of HPV fluctuated with the seasons and that the greatest HPV-positivity rates were in Spring, whereas the lowest were recorded in Autumn (Chen et al., 2019). Nevertheless, other authors reached opposite conclusions regarding similar datasets (Yenidunya et al., 2012; Klebanoff and Turner, 2014). For example, a retrospective study conducted in Turkey revealed that seasonal fluctuations in prevalences of BV, VVC, and TV were not statistically significant (Yenidunya et al., 2012). Furthermore, a longitudinal study of vaginal flora performed in the USA similarly showed that the incidence of BV did not exhibit substantial seasonal variation (Klebanoff and Turner, 2014). According to the findings from our study, we noted no significant effect of seasonality in terms of the prevalences of HR-HPV, LR-HPV, BV, VVC, or TV. Nevertheless, we discovered that there was seasonal change in the incidence of AV.

Most of the extant research indicates a positive correlation between vaginal infections and the acquisition of HPV (Watts et al., 2005; Depuydt et al., 2010; Donders et al., 2013; Feng et al., 2018; Yang et al., 2020; Feng et al., 2023). Recognizing that vaginal infections could induce substantial inflammatory responses that undermined the cervical epithelium’s integrity by creating micro-abrasions, which likely facilitated viral acquisition by providing access to basal keratinocytes, and that these infections produced antimicrobial oxidants that might cause DNA damage in the host, thereby creating a reciprocal relationship where HPV-induced local immune suppression hindered the restoration of a healthy microbiome (Lewis et al., 2013; Chen Z. et al., 2015; Zhang et al., 2026), Watts et al. found that BV (OR, 1.58; 95% CI, 1.49–1.68; *p* < 0.05) and TV (OR, 1.63; 95% CI, 1.49–1.78; *p* < 0.05) infections increased the risk of acquiring HPV infection among American women (Watts et al., 2005). Similarly, a positive correlation between vaginal infections and HPV infection was also reported in Belgium and China (Depuydt et al., 2010; Donders et al., 2013; Feng et al., 2018; Yang et al., 2020; Feng et al., 2023). However, some studies have indicated no correlation between the acquisition of HPV and vaginal infections (Verteramo et al., 2009; Liang et al., 2019; Hu et al., 2021; Liu et al., 2024). Authors of a recent cross-sectional investigation did not uncover any differences in the incidences of VVC (*p* = 0.139) or TV (*p* = 0.105) between HPV-positive and HPV-negative populations (Liang et al., 2019). Furthermore, another cross-sectional study conducted in Italy lacked any correlation between HPV infection and BV (*p* = 0.06), TV (*p* = 0.94) (Verteramo et al., 2009). Similarly, a clinical trial conducted in Jiangsu Province of China showed that the persistence of HPV infection (excluding HPV16 and HPV18) in women was not correlated with prevalent vaginal infections (*p* > 0.05), irrespective of HPV16/18 vaccination status (Hu et al., 2021). A meta-analysis by Liang et al. also showed that the OR for TV did not differ between the HPV-positive and HPV-negative groups (*p* = 0.22) (Liang et al., 2019). We herein demonstrated statistically significant differences in HPV infection rates among patients with BV (OR, 1.95; 95% CI, 1.72–2.21; *p* = 0.000), AV (OR, 1.33; 95% CI, 1.10–1.61; *p* = 0.003), or TV (OR, 1.97; 95% CI, 1.25–3.11; *p* = 0.003); however, rates for women with VVC and those without VVC did not differ (*p* = 0.931). We also ascertained that the risk of HR-HPV infection was higher in patients with BV (OR, 2.04; 95% CI, 1.79–2.33; *p* = 0.000), AV (OR, 1.39; 95% CI, 1.14–1.69; *p* = 0.001), or TV (OR, 1.96; 95% CI, 1.22–3.14; *p* = 0.005); however, HR-HPV infection was not associated with VVC (*p* = 0.639). An noteworthy finding in our analysis was the inverse relationship between HPV infection and intermediate BV, which contrasted with the robust positive link observed with BV. We hypothesized that this might be ascribed to the unstable and transitional characteristics of intermediate BV. HPV infection might expedite the transition from a healthy condition to severe dysbiosis by modifying local mucosal immunity (Cui et al., 2025). Consequently, women infected with HPV might swiftly advance from the intermediate stage to complete BV, resulting in a diminished number of persons in the intermediate group at the time of sampling. The rapid progression hypothesis posited that HPV does not safeguard against intermediate dysbiosis but instead propelled the microbiome towards a more pronounced state of imbalance.

Intriguingly, the previously noted studies exclusively focused only on differences in vaginal infections between HPV-positive and HPV-negative women; therefore, finding a correlation between vaginal infections and specific HPV genotypes requires additional exploration. To the best of our knowledge, only two studies in Tanzania and Kenya have reported an association between vaginal infections and specific HPV genotypes, and there are no pertinent studies on female Chinese cohorts. An analysis of rural Tanzanian women who presented for cervical cancer screening indicated that TV was substantially linked with HPV16 genotype (OR, 6.5; 95% CI, 1.1–37; *p* = 0.04) (Lazenby et al., 2014). Another study conducted on female sex workers in western Kenya revealed significant associations between TV and HPV31 (OR, 2.0; 95% CI, 1.0–3.8; *p* = 0.04), as well as between VVC and HPV16 (OR, 1.9; 95% CI, 1.1–3.3; *p* = 0.03) or HPV53 (OR, 2.0; 95% CI, 1.1–4.0; *p* = 0.03) (Menon et al., 2016). In our investigation of cohorts of Chinese women, we identified the risk of BV infection as higher in patients with HPV45 (OR, 3.33; 95% CI, 1.14–9.75; *p* = 0.037), HPV51 (OR, 1.96; 95% CI, 1.17–3.27; *p* = 0.009), HPV52 (OR, 1.73; 95% CI, 1.25–2.39; *p* = 0.001), HPV58 (OR, 2.14; 95% CI, 1.42–3.21; *p* = 0.001), HPV66 (OR, 2.03; 95% CI, 1.17–3.50; *p* = 0.010), and HPV68 (OR, 2.00; 95% CI, 1.12–3.57; *p* = 0.017). AV also exhibited a higher infection rate in women with HPV16 (OR, 1.97; 95% CI, 1.15–3.39; *p* = 0.012), HPV33 (OR, 3.02; 95% CI, 1.16–7.84; *p* = 0.036), and HPV68 (OR, 2.37; 95% CI, 1.13–5.00; *p* = 0.019); while TV demonstrated a higher infection rate in women with HPV 52 (OR, 3.78; 95% CI, 1.62–8.81; *p* = 0.007). It was worth noting that the association between BV and specific HPV genotypes, particularly HPV16 and HPV18, exhibits significant heterogeneity across different populations. Feng et al. identified a substantial positive connection between BV and HPV16 infection in a study conducted in Northwest China (Feng et al., 2023). Conversely, our data from Jinan (East China) revealed no statistically significant correlation between BV and HPV16 or HPV18, although robust relationships with other high-risk genotypes were seen. This disagreement indicated that the synergistic relationship between vaginal dysbiosis and HPV16/18 may not be ubiquitous but rather contingent upon the population.

There are some limitations to our study. First, our findings were not integrated with cytologic results, hindering us from correlating HPV infection with various cervical abnormalities. Second, we did not document our patients’ personal information. Vaginal microecology is influenced by various factors that include heredity, lifestyle, and hormonal variations that are not entirely regulated. Third, this was a cross-sectional analysis, and therefore, we could not discern a causal relationship between alterations in vaginal microecology and HPV infection. Longitudinal investigations are thus required to allow monitoring of the temporal progression of these infections. Fourth, the vaccination rates among women were not considered, thus precluding the calculation of the vaccine’s specific effect on HPV infection rates.

## Conclusions

In summary, the prevalence and genotypic distribution of HPV infection in Jinan, China, were determined by analyzing 10,104 female patients. While there was no statistically significant difference between HPV infection and VVC, our analysis of the association between HPV infection and vaginal infections revealed that women with BV, AV, TV, or any type of vaginal infection manifested a higher probability of HPV infection. Furthermore, our correlational analysis of disparate HR-HPV genotypes and vaginal infections depicted an elevated incidence of BV in individuals with HPV45, HPV51, HPV52, HPV58, HPV66, and HPV68. AV demonstrated an elevated infection rate in women with HPV16, HPV33, and HPV68; while TV showed an increased infection risk in women with HPV52. We posit that our findings will provide a more robust theoretical framework for the prevention, evaluation, and follow-up of HPV-positive patients with associated infectious illnesses.

## Data Availability

The original contributions presented in the study are included in the article/Supplementary Material. Further inquiries can be directed to the corresponding author.
